# Curcumin as a Natural Therapeutic Agent: A Rapid Review of Potential Clinical Uses and Mechanisms of Action

**DOI:** 10.5812/ijpr-156983

**Published:** 2025-02-04

**Authors:** Mohammad Mohajeri, Reza Momenai, Somayyeh Karami-Mohajeri, Mandana Ohadi, Mohammad Amin Raeisi Estabragh

**Affiliations:** 1 Department of Pharmacology and Toxicology, School of Pharmacy, Kerman University of Medical Sciences, Kerman, Iran; 2 Nimeh Shaaban Hospital, Kerman, Iran; 3 Pharmaceutics Research Center, Institute of Neuropharmacology, Kerman University of Medical Sciences, Kerman, Iran; 4 Student Research Committee, Kerman University of Medical Sciences, Kerman, Iran

**Keywords:** Curcumin, Pharmacological Effects, Therapeutic Properties, Antioxidant, Anti-inflammatory, Anti-cancer

## Abstract

**Context:**

Curcumin, a natural compound derived from the rhizome of the turmeric plant, exhibits various pharmacological and therapeutic effects through distinct cellular and molecular mechanisms.

**Evidence Acquisition:**

Given the therapeutic applications and pharmacological properties of curcumin, it is essential to explore its pharmacological effects for potential use in clinical research. Notably, curcumin demonstrates significant antioxidant, anti-inflammatory, and anti-cancer properties. Importantly, no side effects or specific toxicity have been reported for curcumin.

**Results:**

Curcumin, as a natural compound, can be utilized as a drug supplement in treatment regimens for various diseases. Numerous clinical studies have indicated that curcumin enhances the efficacy of chemotherapy drugs or mitigates their side effects when used concurrently.

**Conclusions:**

This review presents an overview of studies conducted on the pharmacological effects and therapeutic properties of curcumin.

## 1. Context

**Figure 1.**
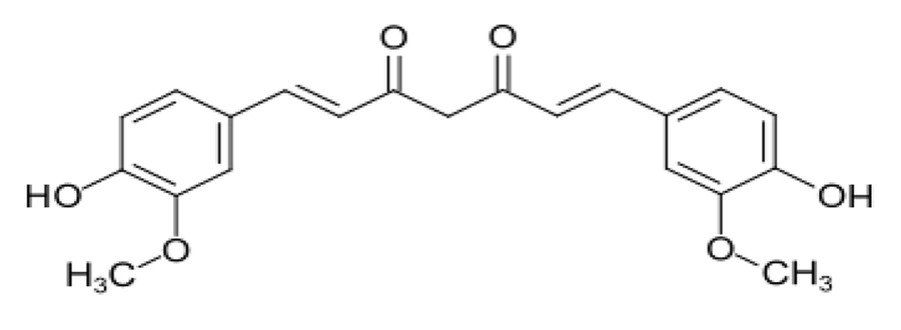
The chemical structure of curcumin

The use of medicinal plants in disease treatment is on the rise. The extraction and analysis of herbal compounds for their effects on various diseases, including cancer, have garnered significant attention (1). Curcumin, chemically known as diferuloylmethane, is an active compound found in the rhizome of the turmeric plant (*Curcuma longa*) (2) (Figure 1). The yellow and golden hue of turmeric is primarily attributed to curcumin, which constitutes approximately 2% to 8% of its compounds, contributing to many of turmeric's properties (3, 4).

Curcumin was first extracted and purified from turmeric in 1815, with its structure identified as diferuloylmethane in 1910 (5). It was determined that curcumin is formed through a bond between two chromophore groups of aryl butene-2-anne (feruloy) and a methylene group (3, 5, 6). Curcumin possesses both phenolic groups and conjugated bonds, rendering it a lipophilic fluorescent substance (1, 2).

Curcumin exhibits extensive pharmacological activities, including antioxidant, anti-inflammatory, antimicrobial, and anti-cancer properties, despite its low intrinsic toxicity (2, 6, 7). Additional activities attributed to curcumin include hypolipidemic effects (8), liver protection (9), inhibition of lipoxygenase (LOX) (10) and cyclooxygenase (COX) (11), protease inhibition (12), free radical scavenging (13), inhibition of lipid peroxidation (14), cholesterol reduction (15), reduction of platelet aggregation (16), reduction of cancer cell proliferation (17), enhancement of food digestion through increased bile flow (18), and modulation of cytokines and other inflammatory factors (19, 20).

Currently, it is believed that an imbalance of inflammatory reactants contributes to many chronic diseases (21, 22). Turmeric, particularly curcumin, has demonstrated a significant anti-inflammatory effect across multiple systems, as evidenced by recent scientific studies. Consequently, turmeric and curcumin are extensively used in the treatment of numerous diseases (23-27).

## 2. Evidence Acquisition

### 2.1. The Main Mechanisms of Curcumin

Curcumin exerts its effects on various cells through multiple cellular pathways and by influencing different receptors and messengers. In the following sections, we will discuss and review some of the mechanisms and molecules involved in the effects of curcumin.

#### 2.1.1. Pre-oxidant and Antioxidant Effects of Curcumin

While pro-oxidants are believed to act as mediators in various diseases, antioxidants are commonly employed to delay or prevent disease progression. Numerous reports indicate that curcumin can function as both a pro-oxidant and an antioxidant (28). Regarding the mechanism of curcumin's pro-oxidant effects: (1) It stimulates the expression of reactive oxygen species (ROS) within cells, playing a crucial role in inducing the cellular anti-proliferative effects of this compound; (2) it binds with thioredoxin reductase (TR), leading to the overproduction of ROS in cancer cells (29-33).

Curcumin exhibits significant antioxidant and free radical scavenging effects in both living and non-living environments (34). This compound protects normal cells from oxidative damage by neutralizing ROS. It is inferred that the antioxidant and scavenging activities of curcumin arise from the phenolic OH groups and the CH_2_ group of the beta-ketone part of the molecule (35). Free radicals are neutralized and inactivated by receiving protons from curcumin or by accepting electrons from this compound (36). Curcumin has also demonstrated the ability to accept electrons and regenerate. In a study conducted by the authors on the oxidation-reduction behavior of curcumin on the surface of a hanging mercury drop electrode and its oxidation-reduction mechanism, it was found that curcumin can readily accept electrons from reducing species and regenerate through a four-electron mechanism (37).

Curcumin also exhibits its antioxidant effects primarily by inhibiting superoxide radicals, hydrogen peroxide, and nitric oxide (38). It has been shown to increase the activity of various antioxidant enzymes, such as catalase (39), superoxide dismutase (40), co-oxygenase (26), and glutathione peroxidase, thereby preventing lipid peroxidation (41). Additionally, curcumin enhances the activity of detoxifying enzymes in the liver and kidneys, protecting normal cells against carcinogenesis processes (9, 15, 33). It also boosts the activity of other enzymes, such as glutathione transferase (42), increases the levels of reduced glutathione and free sulfhydryl groups, and ultimately raises the antioxidant capacity of the living environment (14, 43).

#### 2.1.2. Anti-inflammatory Effects

Curcumin has demonstrated anti-inflammatory properties in numerous studies. Oxidative stress is a major contributor to chronic inflammatory diseases, and antioxidants have been shown to possess anti-inflammatory properties (28, 44). Curcumin exhibits high antioxidant activity, which may underlie its anti-inflammatory effects (45). The anti-inflammatory properties of curcumin are manifested through multiple mechanisms, including the inhibition of nuclear factor-κB (NF-κB) activation, which induces the expression of pro-inflammatory gene products (46-49).

Curcumin modulates the expression of inflammatory enzymes such as cyclooxygenase-2 (COX-2) and inducible nitric oxide synthase (iNOS), both of which play roles in various inflammatory processes (50, 51). Another pro-inflammatory enzyme inhibited by curcumin is 5-lipoxygenase (5-LOX); curcumin inhibits 5-LOX activity by binding to its active site (52). Curcumin reduces the expression of several cell surface molecules that bind to inflammatory mediators (12, 19, 25, 41). It also decreases the expression of C-reactive protein (CRP) and various inflammatory cytokines, including tumor necrosis factor-alpha (TNF-α), interleukin-8 (IL-8), interleukin-6 (IL-6), and chemokines (53, 54). Curcumin inhibits the activity of TNF-α, one of the most important pro-inflammatory mediators (55). Additionally, curcumin inhibits the proliferation and migration of T lymphocytes (56).

#### 2.1.3. Regulatory Effects on Cytokines and Growth Factors

Curcumin has been shown to regulate the cellular activity of cytokines and various growth factors (57). Firstly, curcumin modulates the effect of epidermal growth factor (EGF) by reducing the expression and activity of EGF receptors (58). Secondly, curcumin modulates the activity of human epidermal growth factor receptor 2 (HER2/neu), a growth factor receptor closely associated with breast, lung, kidney, and prostate cancers. Thirdly, curcumin suppresses IL-6 activity by modulating signal transducer and activator of transcription 3 (STAT3) (10, 58, 59). Additionally, curcumin inhibits transforming growth factor-beta 1 (TGF-β1) and reduces the production of several pro-inflammatory cytokines, such as TNF-α and monocyte chemoattractant protein-1 (MCP-1) (60-62).

#### 2.1.4. Roles in Angiogenesis

Angiogenesis is a critical stage in tissue development, playing a vital role in the progression of solid tumors (63). Numerous molecules are involved in angiogenesis, including vascular endothelial growth factor (VEGF), fibroblast growth factor (FGF), COX-2, and TNF-α (64, 65). Firstly, curcumin modulates VEGF expression by inhibiting NF-κB expression and disrupting its signaling role in angiogenesis (66). Secondly, curcumin modulates FGF-dependent angiogenesis (67). Thirdly, curcumin negatively modulates COX-2 expression (68-70). Fourthly, it inhibits the expression and activity of TNF-α. Curcumin also inhibits angiogenesis by reducing the expression of proteins involved in angiogenesis, such as matrix metalloproteinase-2 (MMP-2), matrix metalloproteinase-9 (MMP-9), and protein kinase C-alpha (PKC-α). Based on this evidence, curcumin can inhibit angiogenesis through several pathways (55, 71-73).

**Figure 2.**
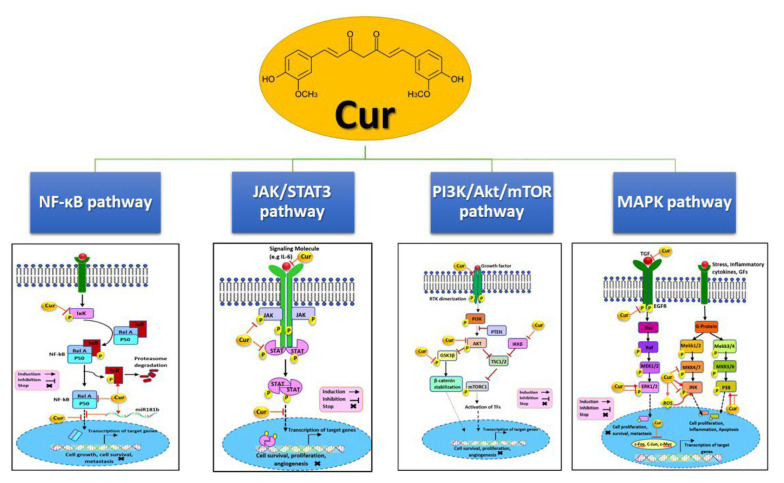
Some pathways that curcumin effects on them, with some changes from the article of Farghadani and Naidu (74).

Figure 2 illustrates several pathways affected by curcumin. Curcumin inhibits the phosphoinositide 3-kinase (PI3K)/AKT/mTOR (PAM) signaling pathway by downregulating IKKβ, AKT, glycogen synthase kinase 3 beta (GSK3β), and HER2, potentially reducing cellular growth, invasion, and metastasis. It also affects the Janus kinase/signal transducer and activator of transcription 3 (JAK/STAT3) pathway by lowering STAT, JAK, and IL-6 levels and preventing STAT translocation into the nucleus, thereby suppressing proliferation and invasion in hormone receptor contexts. Additionally, curcumin modulates the mitogen-activated protein kinase (MAPK) pathway, targeting transforming growth factor (TGF), epidermal growth factor receptor (EGFR), extracellular signal-regulated kinases 1/2 (ERK1/2), mitogen-activated protein kinase kinase 4/7 (MKK4/7), c-Jun N-terminal kinase (JNK), and p38, and reducing nuclear c-Myc, c-Fos, and c-Jun, leading to decreased proliferation, migration, and induced apoptosis. Lastly, curcumin upregulates IκB and miR181b while downregulating NF-κB and IKK, preventing NF-κB nuclear translocation, which in turn inhibits proliferation, survival, metastasis, and angiogenesis (74).

## 3. Results

### 3.1. Potential Clinical Uses of Curcumin and Mechanisms of Action

Considering the mechanisms and cellular pathways discussed above, some of the clinical potentials of curcumin are outlined below.

### 3.1.1. Cancer

Curcumin has demonstrated preventive and therapeutic effects in various cancers (75). The formation or spread of tumors can be prevented or reduced by this compound, as evidenced by numerous studies (76, 77). The cell proliferation cycle is impacted by antiangiogenic effects, induction of apoptosis, and interference (78-80). Curcumin can exhibit its anti-cancer effects through several mechanisms (Table 1). 

**Table 1.** Summary of the Study on the Role of Curcumin in Cellular Signaling Pathways in Different Types of Cancer

Cell Signaling Pathways	Models	Curcumin Administered Doses	Effects	References
**Wnt/β-catenin (µM)**	Human cell line A549	60	Downregulation/inhibition	(81)
**VEGF (mg/kg)**	Nude mice	100	Downregulation/inhibition	(82)
**NOTCH 1 (µM)**	Human lung cancer cell lines	6	Downregulation/inhibition	(83)
**ERK ½ (ng/mL)**	Human NCI-H1975 line	10	Downregulation/inhibition	(84)
**Akt/mTOR (µM)**	Human breast cell lines	10 or 30	Downregulation/inhibition	(85)
**NF-κB (µM)**	Human breast cell lines	20 or 25	Downregulation/inhibition	(86)
**Bcl-2 and Bcl-XL and Autocrine GH (µM)**	T47D human breast cells	20	Downregulation/inhibition	(87)
**MDR-1 (µM)**	MCF-7 breast cancer cell line	1.3	Downregulation/inhibition	(88)
**FEN1 (µM/L)**	MCF-7 breast cancer cell line	0 - 50	Downregulation/inhibition	(89)
**STAT3 and IAP (µM)**	Human GBM stem cells	25	Downregulation/inhibition	(90)
**Platelet-derived growth factor (µM)**	Rat pancreatic stellate cells	25	Downregulation/inhibition	(91)
**PI3 K/Akt (µM)**	Panc-1 human pancreatic cells	20	Downregulation/inhibition	(92)
**IAP (µM)**	Panc-1 human cells	10/50/100	Downregulation/inhibition	(93)
**Cdc20 (µM)**	Patu8988 and Panc-1 human cell lines	10 or 20	Downregulation/inhibition	(94)
**PI3K (µM)**	Human SGC-7901 and BGC-823 cells	10/20/40	Downregulation/inhibition	(95)
**Wnt3 and a/β-catenin/EMT and Bcl-2 (µM)**	Human gastric cell lines	20	Downregulation/inhibition	(96)

Abbreviations: VEGF, vascular endothelial growth factor; NF-κB, nuclear factor-κB; STAT3, signal transducer and activator of transcription 3; PI3K, phosphoinositide 3-kinase.

Firstly, curcumin can inhibit and suppress cell proliferation in a wide range of cancer cells by modulating anti-apoptotic gene products, activating caspases, and stimulating cancer-suppressing genes such as p53 (97, 98). This compound demonstrates its antiangiogenic effects by inhibiting VEGF, angiopoietin I and II, and tyrosine kinase receptors such as Flk-1/KDR (one of the VEGF receptors). Additionally, curcumin activates apoptosis by activating Bcl-2 and Bcl-XL proteins, inducing caspases 3, 8, and 9, releasing cytochrome c, and activating peroxisome proliferator-activated receptor gamma (PPAR-γ) (99-101).

Secondly, curcumin inhibits tumor invasion through the modulation of matrix metalloproteinases (MMPs), cell surface adhesion molecules, AP-1, NF-κB, TNF-α, LOX, COX, chemokines, and growth factors such as EGFR and HER-2, as well as the inhibition of terminal deoxynucleotidyl transferase (TdT) activity and protein tyrosine kinase (102, 103). Exposure of various human gastrointestinal cell lines to curcumin inhibits lipid peroxidation, COX-2 expression, and prostaglandin E2 (PGE2) production, while increasing the level of glutathione S-transferase enzyme (104, 105).

Thirdly, the suppression of angiogenic cytokines such as interleukin-23 (IL-23), IL-6, and interleukin-1 beta (IL-1β) contributes to curcumin's inhibition of angiogenesis in certain tumors (106-108).

Fourthly, curcumin's anti-tumor effects are partly due to its ability to reduce inflammation, given the connection between inflammation and cancer. This compound reduces the production of inflammatory mediators, such as cytokines, COX-2, LOX-2, inducible iNOS, and related cytokines, thereby preventing several types of cancer (109, 110).

Fifthly, curcumin has a chemopreventive effect that can suppress tumor spread, which is one of the possible mechanisms for its action. Its topical application strongly inhibits inflammation caused by 12-O-tetradecanoylphorbol-13-acetate (TPA), cell proliferative hyperplasia, ornithine decarboxylase (ODC) activity, production of ROS, DNA oxidative changes, and papilloma formation (111-113). Since the production of ROS is involved in carcinogenic processes, part of the anti-cancer effects of curcumin is related to its antioxidant and ROS-scavenging effects (29, 35).

Another mechanism for the anticancer effects of curcumin is its involvement in the cell cycle and reduced expression of cyclin-dependent kinases (CDKs). The CDKs are serine-threonine kinases that control cell cycle progression (114). Curcumin also inhibits STAT3 phosphorylation, which is responsible for signaling carcinogenic pathways (115).

### 3.1.2. Diabetes

Curcumin plays a role in the treatment of type II diabetes, a condition characterized by insulin resistance (116) Oxidative stress caused by hyperglycemia, alterations in energy metabolism, and inflammatory mediators significantly contribute to the pathology of diabetes, depletion of cellular antioxidant defense systems, and induction of ROS production (117, 118). Oxidative stress, along with hyperglycemia, impairs cellular, vascular, and neuronal functions. High glucose concentrations induce free radical production through mechanisms involving advanced glycation end products (AGEs), activation of protein kinase C, and the aldose reductase pathway (119).

Another critical factor contributing to increased ROS is TNF, which is linked to obesity and diabetes and is associated with insulin resistance and diabetes complications (120). Since NF-κB and TNF are involved in the induction of insulin resistance, and curcumin can modulate NF-κB activity and TNF expression, curcumin may be effective in reducing the incidence of type II diabetes (119). Curcumin also aids in the management of diabetes (121).

Curcumin exhibits several effects, including proteasome inhibition (a protein complex that regulates the levels of proteins involved in apoptosis), neuroprotective, antioxidant, anti-inflammatory, hypoglycemic, lipid-lowering, and hemoglobin A1c-reducing effects, which collectively slow down or halt the progression of type II diabetes. Table 2 presents various diabetic animal models used to study the effect of curcumin on blood glucose levels.

**Table 2.** Diabetic Animal Models to Study the Effect of Curcumin on Blood Sugar

Curcumin (Route and Dose)	Time of Treatment	Animal Models	References
**Oral, 60 mg/kg⋅BW**	14 days	Wistar rats	(122)
**0.02% curcumin in diet**	42 days	db/db mice	(123)
**Oral, 60 mg/kg⋅BW**	14 days	Wistar rats	(124)
**Oral, 150 mg/kg⋅BW**	42 days	Wistar rats	(125)
**Oral, 80 mg/kg⋅BW**	15 and 60 days	SD rats	(126)
**Oral, 100 mg/kg⋅BW**	28 days; 56 days	SD rats	(127)
**0.5% curcumin in diet**	16 weeks	Wistar rats	(128)
**Oral, 300 mg/kg⋅BW**	56 days	Wistar rats	(129)
**Oral, 80 mg/kg⋅BW**	21 days	Wistar rats	(130)
**I.P., 10 mM**	28 days	Swiss mice	(131)
**Oral, 50 mg/kg⋅BW**	15 days	C57BL/6J mice	(132)

### 3.1.3. Cardiovascular Diseases

Curcumin has been shown to prevent myocardial infarction and other cardiovascular diseases (133). Atherosclerosis, the most common heart disease, involves the buildup of fat, cholesterol, carbohydrate complexes, and fibrin (which causes blood clots) within the inner walls of major arteries, forming plaques. These plaques can completely or partially obstruct a blood vessel and impede arterial blood flow, potentially leading to clot formation on the plaque's surface (134, 135). If such conditions occur and blood flow in the coronary arteries is interrupted, it can result in a heart attack.

Controllable mechanisms of atherosclerotic proliferation include low-density lipoprotein (LDL) oxidation, abnormal platelet aggregation, and inflammation. Curcumin possesses anti-platelet aggregation and antioxidant properties (136, 137). Its ability to control platelet aggregation appears to be directly dependent on the inhibition of thromboxane and the increased activity of prostacyclin (138).

Curcumin is a potent antioxidant and free radical scavenger, reducing cellular damage. It also lowers blood lipid levels, including triglycerides and cholesterol, particularly LDL and very-low-density lipoprotein (VLDL), while increasing high-density lipoprotein (HDL) levels (27, 29, 81-83, 114, 115). This compound reduces the incidence of cardiovascular diseases by inhibiting or preventing oxidative stress processes and exhibiting direct cardioprotective effects (8, 139, 140). Additionally, curcumin can prevent vascular disorders by reducing the calcification levels of the vascular wall (141).

### 3.1.4. Neurodegenerative Disorders

Curcumin has demonstrated neuroprotective effects in neurological diseases such as Alzheimer's disease (AD), dyskinesia, depression, epilepsy, and several other neurological disorders (142). Oxidative stress is one of the mechanisms leading to neuronal damage in the brain. Alzheimer's disease is a progressive disorder associated with cognitive and memory impairments, speech difficulties, and personality changes (143). Although the underlying cause of AD is not fully understood, there is substantial evidence that oxidative stress and impaired protein metabolism contribute to its pathogenesis.

The neuroprotective ability of curcumin has been demonstrated in studies investigating its protective effects against the side effects of high alcohol doses (144). Various research studies have explored the beneficial effects of curcumin on AD and Parkinson's disease using laboratory animals. These studies have shown that curcumin reduces amyloid pathology (145). Curcumin is believed to prevent the onset and progression of AD by exerting beneficial effects on amyloid metabolism, as well as through its anti-inflammatory and antioxidant properties (146).

Given the widespread use of curcumin as a food additive, whose safety has been established in short-term studies, curcumin is considered a promising agent in the treatment and prevention of AD (147, 148). Some studies have demonstrated that curcumin can prevent blood-brain barrier damage, cerebral edema, cerebral circulatory disorders, and tissue and chemical changes in the central nervous system (CNS). These effects are attributed to the antioxidant and anti-inflammatory properties of curcumin (149, 150).

### 3.1.5. Hepatic Fibrosis

Liver fibrosis and cirrhosis, often resulting from chronic liver damage and disorders, pose significant therapeutic challenges worldwide (151). Currently, liver transplantation is the only treatment for end-stage cirrhosis. Oxidative stress and inflammation play crucial roles in the development of alcohol-induced hepatic fibrosis and the metabolism of multiple polyunsaturated fatty acids (PUFAs). Due to its antioxidant and anti-inflammatory properties, curcumin inhibits liver fibrosis (152).

Curcumin is recognized as a potent hepatic anti-fibrosis agent by inducing the expression of MMPs in the liver (153). Overall, curcumin exerts a protective effect on the liver and can prevent MMP activity. This compound also aids in improving liver damage (154). The primary protective and therapeutic effects of curcumin on liver tissue are attributed to its anti-inflammatory, antioxidant, and anti-fibrogenic activities (155).

Curcumin increases the levels of glutathione and superoxide dismutase enzymes in liver tissue, reduces lipid peroxidation, enhances the levels of detoxifying enzymes, boosts the liver's overall antioxidant capacity, and ultimately inhibits the production of ROS (156). Additionally, curcumin can help improve liver damage by lowering serum levels of fat and uric acid (157).

### 3.1.6. AIDS and Psoriasis

Curcumin has recently been shown to inhibit HIV transcription (158). Mazumder reported that curcumin inhibited P24 antigen production and Tat-dependent transcription. They also demonstrated that curcumin inhibited HIV-1 integrase (159). The anti-AIDS effects of curcumin are attributed to two phenyl rings attached to adjacent molecules through hydroxyl groups (158). Curcumin is also considered a moderate inhibitor of HIV-1 and HIV-2 proteases (160). This compound can inhibit HIV-1 through Tat protein degradation (161).

Similar to AIDS, psoriasis involves modulation of immune system activity. Curcumin has beneficial effects on psoriasis in mice through its anti-inflammatory and antioxidant properties (162). In psoriasis, curcumin exhibits its anti-inflammatory effects by reducing the expression of cytokines such as interleukin-17A (IL-17A), interleukin-17F (IL-17F), interleukin-22 (IL-22), IL-6, IL-1β, and TNF-α, and by inhibiting NF-κB activation (163). Curcumin has been shown to improve psoriasis by inhibiting keratinocyte proliferation (164).

Heng et al. (as cited by Thangapazham et al.) revealed that topical treatment with curcumin yielded favorable results, confirmed by immunological, histological, and clinical criteria. According to their findings, these effects of curcumin were associated with the modulation of phosphorylase kinase (PhK) activity in the calcium-calmodulin signaling pathway. This pathway is involved in glycogenolysis and ATP-dependent phosphorylation, providing the necessary energy for cell proliferation and migration (164). The effectiveness of curcumin is partly due to the reduction of PhK levels, as PhK activity is elevated in untreated psoriasis (165).

## 4. Conclusions

The use of curcumin has proven effective in treating various diseases in both animal and human studies. Curcumin is a natural compound with potent anti-inflammatory and antioxidant properties. These properties have led to its application in gastrointestinal and liver diseases, cancer, arthritis, allergies, asthma, atherosclerosis, AD, and hyperglycemia. However, it is important to note that the use of curcumin as a therapeutic agent for the prevention and treatment of diseases requires further clinical trials.
